# Brain stimulation techniques as novel treatment options for insomnia: A systematic review

**DOI:** 10.1111/jsr.13927

**Published:** 2023-05-18

**Authors:** Lukas B. Krone, Kristoffer D. Fehér, Tania Rivero, Ximena Omlin

**Affiliations:** ^1^ University Hospital of Psychiatry and Psychotherapy University of Bern Bern Switzerland; ^2^ Centre for Experimental Neurology University of Bern Bern Switzerland; ^3^ Department of Physiology Anatomy and Genetics, Sir Jules Thorn Sleep and Circadian Neuroscience Institute University of Oxford Oxford UK; ^4^ The Kavli Institute for Nanoscience Discovery University of Oxford Oxford UK; ^5^ Geneva University Hospitals (HUG), Division of Psychiatric Specialties University of Geneva Geneva Switzerland; ^6^ Medical Library University Library of Bern, University of Bern Bern Switzerland

**Keywords:** brain cooling, insomnia disorder, insomnia treatment, transcutaneous auricular vagus nerve stimulation, transcranial direct current stimulation, transcranial magnetic stimulation

## Abstract

Despite the success of cognitive behavioural therapy for insomnia and recent advances in pharmacotherapy, many patients with insomnia do not sufficiently respond to available treatments. This systematic review aims to present the state of science regarding the use of brain stimulation approaches in treating insomnia. To this end, we searched MEDLINE, Embase and PsycINFO from inception to 24 March 2023. We evaluated studies that compared conditions of active stimulation with a control condition or group. Outcome measures included standardized insomnia questionnaires and/or polysomnography in adults with a clinical diagnosis of insomnia. Our search identified 17 controlled trials that met inclusion criteria, and assessed a total of 967 participants using repetitive transcranial magnetic stimulation, transcranial electric stimulation, transcutaneous auricular vagus nerve stimulation or forehead cooling. No trials using other techniques such as deep brain stimulation, vestibular stimulation or auditory stimulation met the inclusion criteria. While several studies report improvements of subjective and objective sleep parameters for different repetitive transcranial magnetic stimulation and transcranial electric stimulation protocols, important methodological limitations and risk of bias limit their interpretability. A forehead cooling study found no significant group differences in the primary endpoints, but better sleep initiation in the active condition. Two transcutaneous auricular vagus nerve stimulation trials found no superiority of active stimulation for most outcome measures. Although modulating sleep through brain stimulation appears feasible, gaps in the prevailing models of sleep physiology and insomnia pathophysiology remain to be filled. Optimized stimulation protocols and proof of superiority over reliable sham conditions are indispensable before brain stimulation becomes a viable treatment option for insomnia.

## 1 INTRODUCTION

The treatment of insomnia has considerably improved in recent years. Cognitive behavioural therapy for insomnia (CBT‐I) represents the first‐line treatment option and has proven effective in various patient populations (Hertenstein et al., 2022; van der Zweerde et al., 2019), even when delivered digitally (Luik et al., 2017). However, about one‐third of patients receiving CBT‐I do not respond to treatment and only about half achieve full remission (Morin et al., 2009). Therapeutic options for non‐responsive patients are limited due to the current lack of safe and effective pharmacotherapies for long‐term insomnia treatment (Riemann et al., 2017). While recent studies indicate that orexin receptor antagonists provide a novel option for long‐term pharmacological intervention, safety data remain inconclusive (De Crescenzo et al., 2022). Therefore, novel treatment approaches are urgently needed.

Animal models have been used to attempt sleep induction via brain stimulation since the early 20th century, when Swiss physiologist Walter Rudolf Hess demonstrated efficacy in cats (Hess, 1929). Now, sleep can be easily initiated in numerous species through the targeted stimulation of select neuronal populations (Weber & Dan, 2016). However, this remote control of sleep requires genetic engineering, injecting viral vectors, and/or implanting electrodes or optic fibres. This limits translation to human therapies. While deep brain stimulation (DBS) can be performed in humans for treatment‐resistant patients with severe neurological and psychiatric conditions, the invasiveness of the method places strong ethical constraints on its use in clinical trials (Lozano et al., 2019).

Alternatively, non‐invasive brain stimulation (NIBS) techniques allow researchers and clinicians to modulate human brain activity in a safe and reversible fashion (Polanía et al., 2018). Some approaches have proven effective in treating certain neurological and mental disorders, which raises hope for wider application (Hyde et al., 2022). In particular, transcranial magnetic stimulation (TMS) and transcranial electric stimulation (tES) demonstrate great practical and cost‐sensitive translational potential. Techniques that indirectly modulate brain activity include transcutaneous auricular vagus nerve stimulation (taVNS), forehead cooling, auditory stimulation and vestibular stimulation. Most of these techniques have been tested in studies aiming to uncover mechanisms of sleep regulation or manipulation in healthy adult participants. Investigations have only recently began interrogating the effects of NIBS techniques on insomnia symptomatology and treatment potential. Such exploration is certainly warranted, with rationale being the demonstrated ability of NIBS to induce oscillatory brain activity, modulate cortical excitability and/or modify network activity, which contributes to systemic hyperarousal in patients with insomnia (Riemann et al., 2010).

Brain stimulation has therefore become a topic of great interest, with applications ranging from basic science interrogation to practical therapeutic translation. Thus, this systematic review presents current evidence and knowledge in the field surrounding the use of brain stimulation approaches for the treatment of insomnia.

## 2 METHODS

**FIGURE 1:**
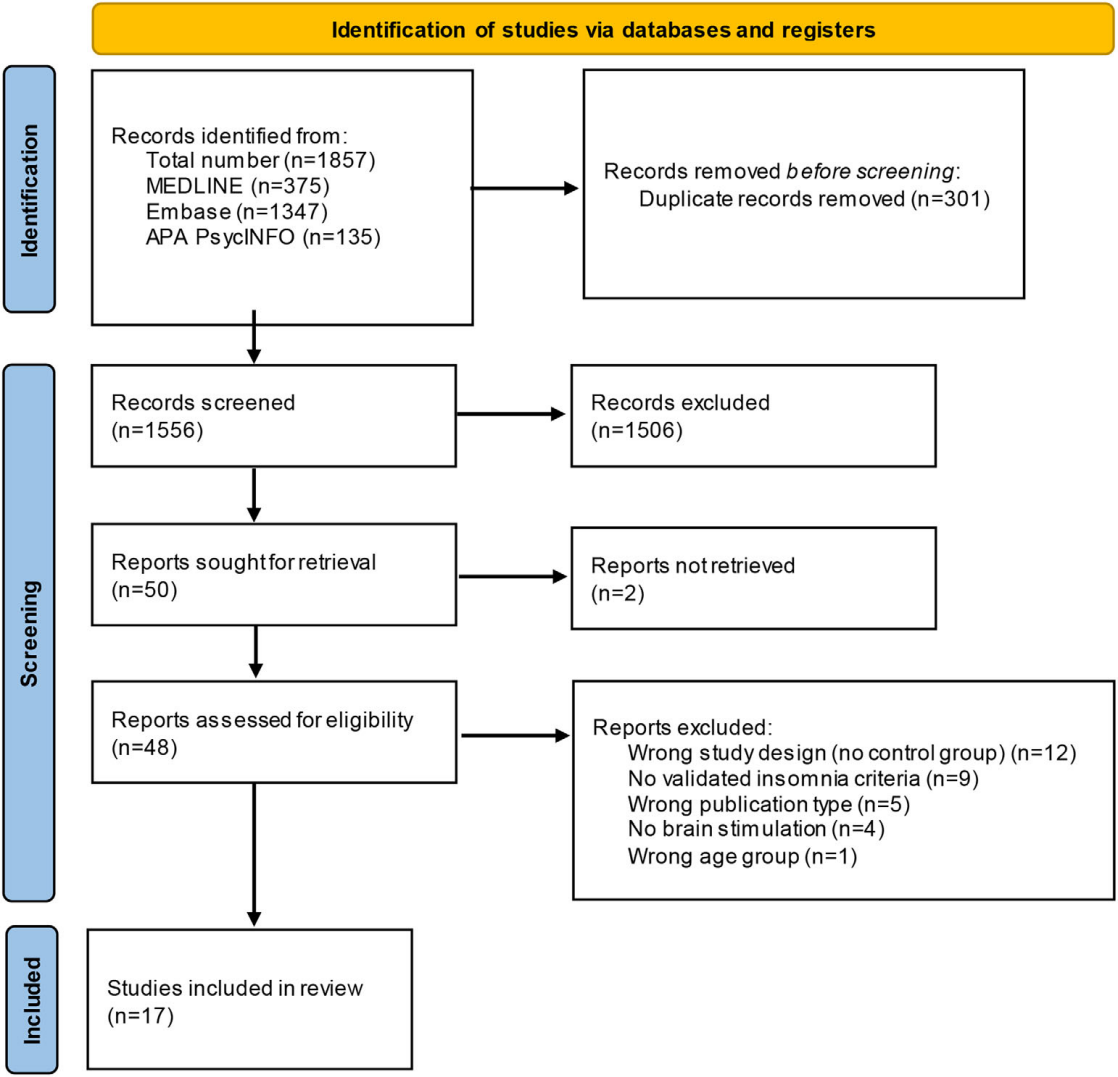
PRISMA 2020 flow diagram [Color figure can be viewed at wileyonlinelibrary.com]

This systematic review was informed by the Cochrane Collaboration handbook and reported according to PRISMA 2020 guidelines (Higgins et al., 2022; Page et al., 2021). We developed the search strategy through analysing key studies. The literature search consisted of a combination of controlled vocabulary terms (e.g. MeSH, EMTREE) and free text terms relating to brain stimulation techniques in insomnia‐related studies (e.g. transcranial electrical stimulation, transcranial magnetic stimulation, auditory stimulation, vagus nerve stimulation, vestibular stimulation and forehead/brain cooling, and technical variations of each). We performed the search in MEDLINE ALL (Ovid), Embase (Ovid) and PsycINFO (Ovid) from inception to 24 March 2023, and the searches were translated appropriately. We considered all publication dates while excluding animal studies. We removed duplicate records using Deduklick – a fully automated de‐duplication software (Borissov et al., 2022). Figure 1 shows the PRISMA flowchart, and the full search strategy is available in the Supplementary File S1.

We divided identified records equally among three members of the research team for review (LBK, KBF, XO), and independently screened the retrieved titles and abstracts for inclusion. We carried out full‐text inspection if the abstract indicated that the study might meet our inclusion criteria. Consensus among three members of the research team (LBK, KBF, XO) determined whether articles would be included for systematic review.

Included papers were peer‐reviewed and written in the English language. The following inclusion criteria were applied such that studies must additionally involve: (1) adult participants (having a minimum age of 18 years and an average age less than 65 years); (2) a population clinically diagnosed with insomnia (based on established diagnostic manuals or assessment by a physician); (3) intervention based on direct or indirect methods of brain stimulation; (4) validated and established sleep outcomes (objective or subjective); (5) a study design with a control group or condition. Exclusion criteria selected against conference abstracts/proceedings, case reports, trial protocols or reviews. We included studies where insomnia was comorbid with anxiety and depression, due to the high degree of overlap between these three conditions, if the investigation explicitly focused on sleep. That is, we excluded studies where insomnia was secondary to or comorbid with other psychiatric or physical health conditions such as pain, brain trauma or neurodegenerative diseases. We furthermore excluded studies that employed a stimulation technique with an undisclosed protocol, which would preclude comparative evaluation and reproduction.

We assessed risk of bias for all studies that met the inclusion criteria using the Cochrane risk of bias tool (RoB 2; Higgins et al., 2022). Two members of the research team (XO, LBK) appraised all papers across five domains of bias: randomization process; deviations from the intended interventions; missing outcome data; measurement of the outcome; and selection of the reported result. The integrated RoB 2 algorithm judged the risk of bias both independently for each domain and across all domains (overall bias) and for each study. Studies were judged to either be at “low” risk of bias, to raise “some concerns”, or to be at “high” risk of bias.

## 3 RESULTS

### 3.1 Search results characterization

Our search yielded 1857 records (see Figure 1 for the PRISMA flow diagram). After removing duplicates, we screened 1556 publications such that 50 articles were considered for full‐text review with 48 being assessed for eligibility. After full‐text review, we selected 17 studies for final inclusion. We identified studies using the following NIBS approaches covered further in this review: (1) TMS (nine studies); (2) tES (five studies), including transcranial direct current stimulation (tDCS; three studies) and transcranial alternating current stimulation (tACS; two studies); (3) taVNS (two studies); and (4) forehead cooling (one study).

Out of all 17 studies, only two classified as having an overall “low” risk of bias: one study using forehead cooling (Roth et al., 2018) and the other employed taVNS (Jiao et al., 2020). Across tES studies, three raised “some concerns” (Frase et al.,  2019; Wang et al., 2020; Zhou et al., 2020) and two were judged to have a “high” risk of bias (Motamedi et al., 2022; Saebipour et al., 2015) due to insufficient blinding procedures or sham protocols. Most TMS studies (eight out of nine) were shown to have a “high” risk of bias (Guo et al., 2023; Jiang et al., 2013; Li et al., 2022; Lu et al., 2022; Pu et al., 2023; Zhang et al., 2018, 2022, 2023), mainly due to participants and assessors being aware of the group allocation (non‐blinded study design), a failure to report methods in sufficient detail or selectively reporting results. The only remaining TMS study (Huang et al., 2018) and the second taVNS study (Wu et al., 2022) raised “some concerns” regarding the risk of bias. The risk of bias assessment for each study and for each domain of the Cochrane tool can be found in Figure S1.

### 3.2 Transcranial magnetic stimulation

**FIGURE 2:**
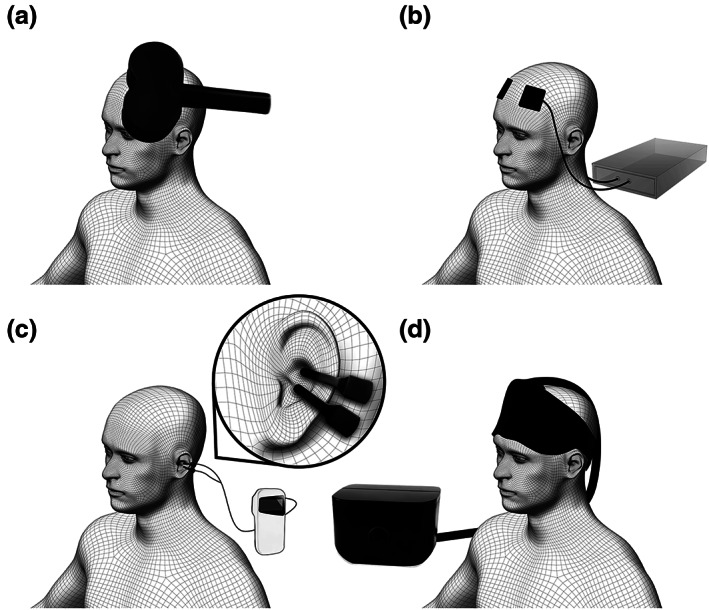
Brain stimulation techniques currently tested as treatment approaches for insomnia: (a) transcranial magnetic stimulation (TMS); (b) transcranial electric stimulation (tES); (c) transcutaneous auricular vagus nerve stimulation (taVNS); (d) forehead cooling

Transcranial magnetic stimulation (Figure 2a) is a NIBS technique that induces localized electrical currents through a magnetic coil based on electromagnetic induction (Valero‐Cabré et al., 2017). It was originally developed as a method to test the function of corticospinal motor pathways (Barker et al., 1985); however, the scope of its application in diagnostic and therapeutic clinical neurosciences has rapidly expanded. Depending on stimulation parameters, researchers can bidirectionally modulate cortical excitability within a few minutes. Typical protocols for repetitive TMS (rTMS) at low frequencies (usually 1 Hz and below) reduce cortical excitability, while high‐frequency protocols (usually 5 Hz and above) increase cortical excitability (Valero‐Cabré et al., 2017). Based on the assumption that systemic hyperarousal in patients with insomnia might be rooted in cortical hyperactivity (Riemann et al., 2010), most TMS studies have attempted to improve the sleep of patients with insomnia using low‐frequency stimulation.

Four double‐blinded randomized–controlled trials tested active 1‐Hz rTMS of the left dorsolateral prefrontal cortex (DLPFC) against a sham rTMS condition in patients with insomnia (Guo et al., 2023; Li et al., 2022; Zhang et al., 2022; Zhang et al., 2023). One study randomized 40 patients with insomnia to real or sham rTMS and found a significantly greater reduction of the Insomnia Severity Index (ISI) in the treatment group (Zhang et al., 2022). However, this investigation is methodologically limited as the TMS therapist was not blinded and, despite mental illness being listed as an exclusion criterion, patients received conceivably confounding hypnotic and antidepressant pharmacotherapy. The remaining three studies were conducted by researchers from Xidian University, and assessed subjective questionnaire and objective polysomnography (PSG) sleep parameters in patients with insomnia (Guo et al., 2023; Li et al., 2022; Zhang et al., 2023). All three report improvements across all main outcome parameters in the treatment group, but not in the control group, in samples of 44, 50 and 60 patients with insomnia, respectively. However, the studies demonstrate shared methodological issues such as a lack of detailed statistical procedures disclosure, high drop‐out and exclusion rates, and a lack of several relevant PSG parameters, such as total sleep time (TST), the duration of rapid eye movement (REM) and non‐REM (NREM) sleep, and the amount of time spent in NREM stages other than NREM stage 3.

Three further studies compared 1‐Hz rTMS treatments of different cortical regions against or in addition to alternative treatment approaches (Jiang et al., 2013; Lu et al., 2022; Zhang et al., 2018). An initial study in 135 patients with insomnia compared rTMS of the right DLPFC with 2 mg estazolam medication and with cognitive behavioural psychotherapy over 2 weeks (Jiang et al., 2013). Each of the three groups had 40 patients who completed the study, and demonstrated a significant improvement across all subjective and objective sleep parameters. rTMS elicited the greatest improvement according to the Pittsburgh Sleep Quality Index (PSQI) and some PSG parameters such as REM sleep and NREM stage 3. The authors also report profound changes in several hypothalamic–pituitary–adrenal and hypothalamic–pituitary–thyroid axes hormone levels, which were unique to medication and rTMS groups. Though surprising and exciting, these results must be interpreted with caution as the study was not blinded and reported effects notably exceed those observed in previous well‐controlled experiments (Mitchell et al., 2019; Winkler et al., 2014). Similar methodological issues are present in a study comparing pharmacotherapy against a combined treatment approach (pharmacotherapy plus rTMS of the right DLPFC over 4 weeks; Lu et al., 2022). The authors found no significant differences in the improvement of PSQI and ISI scores between treatment conditions in this small sample of 24 patients with insomnia, yet reported a trend towards the added benefit of rTMS application. Another study combined acupuncture with real or sham rTMS of the left prefrontal cortex (PFC) in 78 patients with insomnia, which was interrogated due to literature suggesting that acupuncture therapy might benefit insomnia treatment (Zhang et al., 2018). The authors report a significantly greater reduction of ISI scores and improvement of self‐reported sleep efficiency in the combined treatment group; however, no group differences for other measures such as the PSQI, self‐reported TST, sleep‐onset latency (SOL) and wake after sleep onset (WASO) were reported. Interpretation is again restricted by a limited description of applied statistical methods.

Two studies investigated the effects of very different rTMS protocols in patients with insomnia with comorbid general anxiety disorder (GAD; Huang et al., 2018) or depression (Pu et al., 2023). Huang and colleagues randomized 36 patients with comorbid GAD and insomnia to 1‐Hz rTMS or sham rTMS of the right parietal cortex (Huang et al., 2018). This small study reports significant improvements of anxiety, insomnia and depression scores in the active but not in the sham TMS group. In contrast to this low‐frequency rTMS application, Pu and colleagues compared high‐frequency 10‐Hz rTMS of the left DLPFC with sham rTMS in 100 patients with comorbid depression. They report improvements in all subjective and objective sleep parameters, including depression scores, and increased blood serum levels of brain‐derived neurotrophic factor and norepinephrine (Pu et al., 2023). However, the reliability of these findings must be considered alongside limitations, including an unblinded study design. The results of both studies may furthermore be confounded by having permitted participants with psychiatric conditions to take variable doses of short‐acting benzodiazepines (Huang et al., 2018) and agomelatine (Pu et al., 2023).

All nine aforementioned studies performed rTMS during the day and over several days (Table 1). Cortical targets typically involved the left DLPFC/PFC, though two studies stimulated the right DLPFC and one the right parietal lobe. Eight out of nine studies report an improvement in sleep parameters when comparing active with control conditions, with the other one study reporting a present yet non‐significant trend towards rTMS benefit. This trend aligns with other studies that were excluded from systematic review because pre–post analysis was conducted without a control group or condition (Antczak et al., 2017; Feng et al., 2019; Qi et al., 2022; Shi et al., 2021; Song et al., 2019; Wu et al., 2021). However, positive results may be notably compromised by a lack of blinding, insufficiently detailed technical method descriptions, inconsistent statistical analyses, selective results presentation, unclear comorbidity status reporting, and possible confounding effects due to administered medication. These considerable limitations lead to a high risk of bias in eight out of nine studies, and to some concerns of bias in one study (Figure S1).

**TABLE 1:** Characteristics and results of the included primary studies

TMS						
Study	Patients^a^ *N* (gender distribution) Age Insomnia criteria Comorbidities	Study design^b^	Method	Protocol Intensity, pulses/train, *N* trains, train interval, sessions	Stimulation location^c^	Main results^d^ • Subjective outcome measure(s) (PSQI and/or ISI) • PSG (TST, SE, SOL, additional parameters if available) • Further outcome measure(s) (subjective or objective insomnia and sleep related)
Jiang et al. (2013)	*N* (all included) = 135 (45 (24f 21m) rTMS, 45 (26f 19m) medication, 45 (25f 20m) psychotherapy) *N* (completers) = 120 (40 rTMS, 40 medication, 40 psychotherapy) Age (rTMS, medication, psychotherapy; mean ± SD): 48.31 ± 8.45 years, 48.11 ± 7.51 years, 47.02 ± 6.33 years Insomnia criteria: DSM‐4. Additional sleep criteria: PSQI > 6, SE ≤ 80%, SOL ≥ 30 min, TST < 6 hr	Double‐blind, randomized–controlled trial. rTMS versus medication (2 mg estazolam each night) versus psychotherapy (CBT‐I) Sham condition: n.a.	1‐Hz rTMS	80% RMT 30 pulses/train × 60 trains, 2‐s inter‐train‐interval, daily sessions for 2 weeks.	rDLPFC	• PSQI: interaction effect: n.a.; effect group: n.a. Baseline versus post‐rTMS: ↓ Values (baseline, post; mean ± SD): rTMS: 15.6 ± 4.61, 8.02 ± 2.53 medication: 14.2 ± 6.73, 9.37 ± 3.09 psychotherapy: 13.6 ± 5.56, 11.45 ± 6.14 • PSG: interaction effect: n.a.; effect group: n.a. Baseline versus post: rTMS: TST ↑, SE ↑, SL ↓, N1 ↓, N2 ↑, N3↑, REM ↑ • Further outcome measures: rTMS: low relapse and recurrence rates (3 months after treatment)
Huang et al. (2018)	*N* (all included/completers) = 36 (18 (9f 9m) active, 18 (9f 9m) sham) Age (active, sham; mean ± SD, permitted range): 44.94 ± 11.64, 45.22 ± 10.85, 18–65 years Insomnia criteria: DSM‐4. Additional sleep criteria: PSQI ≥ 7 Comorbidities: GAD. Criteria: HRSA ≥ 14, HRSD‐24 < 20	Double‐blind, randomized–controlled trial. Sham condition: sham coil	1‐Hz rTMS	90% RMT 500 pulses/train × 3 trains, 10‐min inter‐train‐interval, daily sessions over 10 consecutive days.	Right parietal lobe (P4)	• PSQI: interaction effect:*;effect group: post ↓, 2‐week follow‐up ↓, 1 month follow‐up ↓ Values (baseline, post, 2‐week follow‐up, 1 month follow‐up; mean ± SD): active: 12.61 ± 2.85, 7.06 ± 2.75, 7.22 ± 2.53, 7.28 ± 3.37 sham: 13.06 ± 4.26, 11.44 ± 4.13, 11.83 ± 3.45, 11.56 ± 3.82 • PSG: n.a. • Further outcome measures: Significantly greater improvement of HRSA anxiety scores in active group. Significantly greater improvement of HRSD‐24 depression scores in active group.
Zhang et al. (2018)	*N* (all included) = 78 (40 active, 38 sham) *N* (completers) = 75 (38 (34f 4m) active, 37 (33f 4m) sham) Age (active, sham, permitted range): 51.3 ± 8.7, 49.8 ± 9.2, 18–60 years Insomnia criteria: modified DSM‐5 criteria. Additional sleep criteria: sleeping disorder > 1 month, ≥ 3× per week, PSQI score 7–15	Double‐blind, randomized–controlled trial. Sham condition: acupuncture plus sham rTMS (sham coil)	1‐Hz rTMS plus acupuncture	100% RMT continuous stimulation for 30 min, 1200 total pulses per session, 3 days per week for 4 weeks.	Left PFC	• ISI: interaction effect: n.a.; main effect group* Values (baseline, post, 2‐week follow‐up; mean ± SD): active: 18.7 ± 2.6, 13.8 ± 6.0, 14.8 ± 2.5 sham: 17.8 ± 2.4, 14.7 ± 4.2, 15.1 ± 5.4 • PSQI: interaction effect: n.a.; main effect group: n.s. Values (baseline, post‐treatment, 2‐week follow‐up; mean ± SD): active: 12.6 ± 2.7, 10.9 ± 3.8, 11.3 ± 3.6 sham: 12.2 ± 3.4, 11.4 ± 3.4, 12.0 ± 3.7 • PSG: n.a. • Further outcome measures: Additional subjective sleep parameters (based on sleep diary): TST: interaction effect: n.a.; main effect group: n.s. SOL: interaction effect: n.a.; main effect group: n.s. WASO: interaction effect: n.a.; main effect group: n.s. SE: interaction effect: n.a.; main effect group: ↑
Zhang et al. (2022)	*N* (all included) = 40 (20 (12f 8m) active, 20 (13f 7m) sham) *N* (completers) = 35 (17 active, 18 sham) Age (active, sham, permitted range): 51.78 ± 11.22 years, 50.45 ± 11.20 years, 18–65 years Insomnia criteria: DSM‐5. Additional sleep criteria: ISI > 7	Double‐blind, randomized–controlled trial. Note: However, therapist using TMS was not blinded, hence in fact single‐blind. Sham condition: sham rTMS (no details)	1‐Hz rTMS	90% RMT 20 pulses/train × 9 trains, 20–30‐s inter‐train‐interval, daily sessions for 14 days.	lDLPFC	• ISI: interaction effect:*; effect group: ↓ active baseline versus post: ↓ • PSG: n.a.
Li et al. (2022)	*N* (all included) = 44 (28f 16m), *N* (completers) = 35 (17 active, 18 sham) Age (all included; permitted range): 43.8 ± 10.3 years, 18–65 years Insomnia criteria: DSM‐5	Double‐blind, randomized–controlled trial. Sham condition: coil 90° turned away from skull	1‐Hz rTMS	80% RMT 10 pulses/train × 150 trains, 5 days per week for 4 weeks.	lDLPFC	• PSQI: interaction effect:*; effect group: n.a. active baseline versus post: ↓ • ISI: interaction effect:*; effect group: n.a. active baseline versus post: ↓ • PSG: N3: interaction effect:*; effect group: n.a. active baseline versus post: ↑ SE: interaction effect:*; effect group: n.a. active baseline versus post: ↑ SOL: interaction effect:*; effect group: n.a. active baseline versus post: ↓
Lu et al. (2022)	*N* (all included/completers) = 24 (12 (9f 3m) active, 12 (7f 5m) sham) Age (active, sham; median ± IQR, permitted range): 53.5 ± 8.0 years, 57 ± 16 years, 45–75 years Insomnia criteria: DSM‐5	Open‐label, randomized–controlled trial. Sham condition: pharmacotherapy only, without TMS	1‐Hz rTMS plus pharmacotherapy	90% RMT 30 pulses/train × 40 trains, 8‐s inter‐train‐interval 5 days per week for 4 weeks.	rDLPFC	• PSQI: interaction effect: n.a.; effect group: n.s. Values (baseline, post; median ± IQR): drug only: 17.0 ± 5.0, 13.0 ± 3.5 drug + rTMS: 15.0 ± 3.5, 8.0 ± 5.5 • ISI: interaction effect: n.a.; effect group: n.s. Values (baseline, post; median ± IQR): drug only: 15.5 ± 7.75, 9.0 ± 4.25 drug + rTMS: 14.5 ± 4.5, 5.0 ± 3.25 • PSG: n.a.
Guo et al. (2023)	*N* (all included) = 60 *N* (completers) = 58 (28f 30m) Age (completers; mean ± SD, permitted range): 43.93 ± 10.93 years, 18–65 years Insomnia criteria: DSM‐5. Additional sleep criteria: PSQI score ≥ 5, ISI score ≥ 8, ESS score ≤ 11, BDI score ≤ 20, BAI score ≤ 45	Double‐blind, randomized–controlled trial. Sham condition: coil 90° turned away from skull	1‐Hz rTMS	80% RMT 10 pulses/train × 150 trains, 2‐s inter‐train‐interval, 5 days per week for 4 weeks.	lDLPFC	• PSQI: interaction effect:*; effect group: n.a. active baseline versus post: ↓ • ISI: interaction effect:*; effect group: n.a. active baseline versus post: ↓ • PSG (*N* = 47, 23 active, 24 sham): N3: interaction effect:*; effect group: n.a. active baseline versus post: ↑ SE: interaction effect:* effect group: n.a. active baseline versus post: ↑ SOL: interaction effect:*; effect group: n.a. active baseline versus post: ↓
Zhang et al. (2023)	*N* (all included) = 50 *N* (completers) = 41 (17 (13f 4m) active, 24 (17f 7m) sham) Age (active, sham; mean ± SEM, permitted range): 45.65 ± 2.57 years, 44.9 ± 2.18 years, 18–65 years Insomnia criteria: DSM‐5. Additional sleep criteria: PSQI score ≥ 5, ISI score ≥ 8	Double‐blind, randomized–controlled trial. Sham condition: coil 90° turned away from skull	1‐Hz rTMS	80% RMT 10 pulses/train × 150, 2‐s inter‐train‐interval, 5 days per week for 4 weeks.	lDLPFC	• PSQI: interaction effect:*; effect group: n.a. Values (baseline, post; mean ± SEM) active: 13.18 ± 0.77, 8.06 ± 0.70 sham: 11.33 ± 0.35, 11.00 ± 0.54 • ISI: interaction effect:*; effect group: n.a. Values (baseline, post; mean ± SEM) active: 16.41 ± 0.99, 9.47 ± 0.93 sham: 12.83 ± 0.57, 11.96 ± 0.68 • PSG: interaction effect: N3*; SE*; SOL*; effect group: n.a active baseline versus post: N3 ↑, SE ↑, SOL ↓
Pu et al. (2023)	*N* (all included) = 100 (50 (31f 19m) active, 50 (28f 22m) sham) *N* (completers) = 82 (42 active, 40 sham) Age (active, sham; mean ± SD, range): 35.24 ± 5.12 years, 21–65 years, 33.97 ± 4.74 years, 23–62 years Insomnia criteria: ICD‐10. Additional sleep criteria: PSQI score > 7 Comorbidities: mild or moderate depressive episode. Criteria: HAMD score: 7–23	Open‐label, randomized–controlled trial. Sham condition: agomelatine plus sham rTMS (no details)	10‐Hz rTMS plus agomelatine, at different doses monitored by clinical response and side‐effects (25–75 mg per day)	120% RMT 80 pulses/train × 25 trains, 26‐s inter‐train‐interval, 5 days per week for 4 weeks.	lDLPFC	• PSQI: interaction effect: week 4 n.a., week 8*; effect group: week 4 ↓, week 8 ↓ Values (week 0, 4, 8; mean ± SD): active: 14.62 ± 3.84, 7.06 ± 2.87, 4.38 ± 1.24 sham: 14.24 ± 3.67, 8.68 ± 2.11, 6.72 ± 1.53 • PSG: TST: interaction effect: week 8*; effect group: week 8 ↑ Values (week 0, 8; mean ± SD): active: 343.41 ± 69.65, 422.33 ± 71.57 sham: 331.57 ± 68.38, 385.41 ± 69.65 SE (W0, W8): interaction effect: week 8*; effect group: week 8 ↑ Values (week 0, 8; mean ± SD): active: 70.78 ± 12.58, 84.83 ± 14.26 sham: 68.47 ± 11.94, 75.32 ± 13.08 SOL (W0, W8): interaction effect: week 8*; effect group: week 8 ↓ Values (week 0, 8; mean ± SD): active: 61.32 ± 10.55, 24.47 ± 5.71 sham: 58.28 ± 11.17, 29.52 ± 6.36 • Further outcome measures: HAMD‐17: interaction effect: week 4 n.a., week 8*; effect group: week 2 ↓, week 4 ↓, week 8 ↓ Additional PSG parameters: awakening time: interaction effect: week 8;* effect group: week 8 ↓ Micro‐awakenings: interaction effect: week 8*; effect group: week 8 ↓ N1: interaction effect: week 8*; effect group: week 8 ↓ N3: interaction effect: week 8*; effect group: week 8 ↑
tES						
Study	Patients^a^ *N* (gender distribution) Age Insomnia criteria Comorbidities	Study design^b^	Method	Protocol Intensity, duration, frequency (if applicable), time of stimulation	Stimulation location^c^ Polarity (location): electrode dimensions	Main results^d^ • Subjective outcome measure(s) (PSQI and/or ISI) • PSG (TST, SE, SOL, additional parameters if available) • Further outcome measure(s) (subjective or objective insomnia and sleep related)
Saebipour et al. (2015)	*N* (all included) = 7 *N* (completers) = 6 (2f 4m) Age: 34 ± 7 years Insomnia criteria: psychiatrist diagnosed insomnia (diagnostic tool not specified)	Single‐blind, within‐subject repeated‐measures, randomized. Sham condition: pseudostimulation (same procedure and set up as active; no further details provided)	toDCS	0.26 mA, ca. 25 min, 0.75 Hz, during N2 or N3 sleep at beginning of the night	A(F3, F4): 8 mm ⌀ C(M1, M2): 8 mm ⌀	• PSQI or ISI: n.a. ISI at baseline: 19 ± 3 • PSG: SOL: interaction effect: n.a.; effect condition: n.s. TST: interaction effect: n.a.; effect condition: n.s. SE: ↑ 9 ± 7% • Further outcome measures: N3: interaction effect: n.a.; effect group: ↑ N1: interaction effect: n.a.; effect group: ↓ N1 + wake: interaction effect: n.a.; effect group: ↓ N2 → N3: interaction effect: n.a.; effect group: ↑ N2 → wake: interaction effect: n.a.; effect group: ↓
Frase et al. (2019)	*N* (all included/completers) = 19 (13f 6m) Age (all included; mean ± SD, range): 43.8 ± 15.1 years, 20–60 years Insomnia criteria: ICD‐10	Single‐blind, within‐subject repeated‐measures, randomized (anodal, cathodal, sham). Sham condition: 30 s fade in ramp up to 1 mA immediate 30 s fade out at beginning and end of sham condition	tDCS	Anodal: 1 mA, 2 × 13 min (20‐min inter‐stimulation‐interval) Cathodal: 1 mA, 2 × 9 min (20‐min inter‐stimulation‐interval), prior to sleep	Target (Fp1, Fp2): 5 × 7 cm Return (P3, P4): 10 × 10 cm	• PSQI or ISI: n.a. PSQI at baseline: 9.4 ± 2.9 • PSG: TST: interaction effect: n.a.; effect condition: n.s. Values (sham, anodal, cathodal; mean ± SD): TST: 389.6 ± 58.3; 396.3 ± 43.6; 393.2 ± 38.7 SOL: interaction effect: n.a.; effect condition: n.s. Values (sham, anodal, cathodal; mean ± SD): SOL: 19.0 ± 18.3; 20.3 ± 14.9; 23.9 ± 22.08 SE: interaction effect: n.a.; effect condition: n.s. Values (sham, anodal, cathodal; mean ± SD): SE: 81.1 ± 12.2; 82.7 ± 9.0; 81.7 ± 8.0 • Further outcome measures: SFA (subjective sleep questionnaire): interaction effect: n.a.; effect condition: n.s.
Zhou et al. (2020)	*N* (all included/completers) = 90 (47 active (31f 16m), 43 sham (29f 14m)) Age (active, sham; mean ± SD, permitted range): 43.91 ± 11.20 years, 40.45 ± 8.31 years, 18–65 years Insomnia criteria: ICD‐10 Comorbidities: depression. Criteria: DSM‐5 and SDS: ≥ 50	Double‐blind, randomized–controlled trial. Sham condition: switch off after 30 s of stimulation	tDCS	2 mA, 20 daily sessions, 30 min, daytime (not further specified)	A(lDLPFC): 5 × 5 cm C(rDLPFC): 5 × 5 cm	• PSQI: interaction effect: n.s.; effect group: n.s. Values (baseline, post; mean ± SD): sham: 13.26 ± 2.91, 7.09 ± 3.71 active: 14.32 ± 3.79, 6.55 ± 2.85 • PSG TST: interaction effect:*; effect group: n.s. Values (baseline, post; mean ± SD): sham: 397.54 ± 54.94, 449.60 ± 27.53 active: 381.16 ± 42.38, 468.75 ± 45.89 SE: interaction effect:*; effect group: n.s. Values (baseline, post; mean ± SD): sham: 75.32 ± 6.59, 76.24 ± 7.79 active: 73.04 ± 6.98, 81.26 ± 12.71 SOL: interaction effect:*; effect group:* Values (baseline, post; mean ± SD): sham: 16.04 ± 2.48, 14.72 ± 2.76 active: 17.76 ± 4.89, 14.94 ± 2.77 • Further outcome measures: Significantly greater improvement of SDS and SAS scores in active group.
Wang et al. (2020)	*N* (all included) = 62 (31 (24 f 7m) active, 31 (23f 8m) sham) *N* (completers) = 60 (30 active, 30 sham) Age (active, sham; mean ± SD): 52.5 ± 10.7 years, 55.3 ± 8.0 years Insomnia criteria: DSM‐4 or ICD‐10; additional sleep criteria: PSQI > 8; PSQI component 7 (daytime dysfunction) ≥ 2	Double‐blind, randomized–controlled trial. Sham condition: no stimulation	tACS	15 mA, 20 daily sessions, 40 min, 77.5 Hz, fixed day time (not further specified)	Target (Fpz, Fp1, Fp2): 4.45 × 9.53 cm Return (M1, M2): 3.18 × 3.81 cm	• PSQI: 4‐week follow‐up interaction effect: n.a.; effect group* Values (baseline, end of intervention, follow‐up; mean, 95% CI): sham: 13.23 (12.44, 14.02), 8.43 (6.98, 9.89), 9.93 (8.42, 11.44) active: 13.13 (12.53, 13.74), 5.93 (4.47, 7.39), 6.73 (5.05, 8.41) • PSG: n.a.
Motamedi et al. (2022)	*N* (all included) = 12 *N* (completers) = 9 (8f 1m) Age (completers; mean: 50.2 years) Insomnia criteria: DSM‐5	Single‐blind, within‐subject repeated‐measures, randomized. Sham condition: same procedure and set up but no stimulation applied	tACS	0.75 mA, 0.75 Hz, 5‐min stimulation upon lights‐off, 1‐min break. If the patient had not entered sleep: another 5 min of stimulation, 1‐min break. Cycle repeated until sleep observed in EEG, as well as upon > 1 min arousal during the night	A(F3, F4): Soterix HD electrode, 1.2 cm external ⌀ C(M1, M2): Soterix HD electrode, 1.2 cm external ⌀	• PSQI: interaction effect: n.a.; effect condition: n.a. active baseline versus post: ↓ Values (baseline, post; mean ± SD): entire sample:14.4 ± 2.9, 8.2 ± 4.3 • ISI: interaction effect: n.a.; effect condition: n.a. active baseline versus post: n.s. Values (baseline, post; mean ± SD): entire sample: 17.9 ± 4.0, 16.9 ± 6.5 • PSG SOL: interaction effect: n.a.; effect condition: n.s. Values (sham post, active post; mean ± SD): 39.9 ± 56.3, 22.2 ± 25.8 SE: interaction effect: n.a.; effect condition: n.s. Values (sham post, active post; mean ± SD): 75.8 ± 19.5, 81.5 ± 10.6
Forehead cooling						
Study	Patients^a^ *N* (gender distribution) Age Insomnia criteria Comorbidities	Study design^b^	Method	Protocol Intensity, duration, time of stimulation	Stimulation location^c^	Main results^d^ • Subjective outcome measure(s) (PSQI and/or ISI) • PSG (TST, SE, SOL, additional parameters if available) • Further outcome measure(s) (subjective or objective insomnia and sleep related)
Roth et al. (2018)	*N* (all included) = 106 (54 (37f 17m) active, 52 (37f 15m) sham) *N* (completers) = 100 (51 active, 49 sham) Age (active, sham, permitted range): 49.9 ± 13.8 years, 50.3 ± 15.4 years, ≥ 22 years Insomnia criteria: DSM‐4. Additional sleep criteria: ISI score > 14, SE ≤ 85% (based on sleep diary and PSG), SOL ≥ 15 min (based on PSG)	Double‐blind, randomized–controlled trial. Sham condition: “vestibular sleep system” (credible device control, sham condition)	Forehead cooling	14–16°C for 8 hr bedtime. Device applied 60 min prior to bedtime at 30°C. Temperature reduction to 15°C over 30 min. One‐time opportunity to self‐adjust temperature before bedtime.	Forehead over the area of the frontal cortex.	• ISI: n.a. Values (active baseline, sham baseline; mean ± SD): 20.5 ± 3.3, 20.4 ± 3.5 • PSG (*N* = 103 (53 active, 50 sham)): TST: interaction effect: n.a.; effect group: n.s. Values (baseline, post; mean ± SD): active: 327.7 ± 50.8, 385.4 ± 45.8 sham: 338.0 ± 47.9, 387.1 ± 46.2 SE: interaction effect: n.a.; effect group: n.s. Values (baseline, post; mean ± SD): active: 68.3 ± 10.6, 80.3 ± 9.5 sham: 70.4 ± 10.0, 80.7 ± 9.6 SOL, absolute and relative change: interaction effect: n.a.; effect group: ↓ Values (baseline, post; mean ± SD): active: 49.2 ± 29.8, 21.9 ± 19.8 sham: 41.7 ± 27.5, 31.9 ± 27.9 • Further outcome measures (*N* = 103 (53 active, 50 sham)): Persistent sleep latency, relative change: interaction effect: n.a.; effect group: ↓ N1 latency, absolute and relative change: interaction effect: n.a.; effect group: ↓ N2 latency, absolute and relative change: interaction effect: n.a.; effect group: n.s. N3 latency, absolute and relative change: interaction effect: n.a.; effect group: n.s. WASO: interaction effect: n.a.; effect group: n.s. Awakenings: interaction effect: n.a.; effect group: n.s. Amount of sleep in the first hour of the night: interaction effect: n.a.; effect group: ↑
aVNS						
Study	Patients^a^ *N* (gender distribution) Age Insomnia criteria Comorbidities	Study design^b^	Method	Protocol Intensity, frequency, duration, time of stimulation	Stimulation location^c^	Main results^d^ • Subjective outcome measure(s) (PSQI and/or ISI) • PSG (TST, SE, SOL, additional parameters if available) • Further outcome measure(s) (subjective or objective insomnia and sleep related)
Jiao et al. (2020)	*N* (all included) = 72 (36 (31f 5m) active, 36 (29f 7m) sham) *N* (completers) = 63 (31 active, 32 sham) Age (active, sham; mean ± SD, permitted range): 47.67 ± 12.0 years, 50.60 ± 12.0 years, 18–70 years Insomnia criteria: DSM‐5	Double‐blind, randomized–controlled trial. Sham condition: transcutaneous non‐vagus nerve stimulation	taVNS	Intensity adjusted by participants, repeated protocol of 20 Hz for 10 s and 4 Hz for 5 s, for 30 min per session, 2 sessions per day, 5 days per week for 2 weeks, applied in the morning and about half an hour before bedtime.	Auricular concha innervated by afferent auricular branch of the vagus nerve (ABVN)	• PSQI: interaction effect: n.a.; effect group: n.s. Values (baseline, week 4): active: 14.5 ± 3.2 (mean ± SD), 8.8 (4.1–14.0) (mean (95% CI)) sham: 14.5 ± 2.7 (mean ± SD), 10.0 (5.0–18.9) (mean (95% CI)) • PSG: n.a. • Further outcome measures: ESS: interaction effect: n.a.; effect group: n.s. FSS: interaction effect: n.a.; effect group: n.s. HAMD‐17: interaction effect: n.a.; effect group: n.s. HAMA‐17: interaction effect: n.a.; effect group: n.s. SF‐36: interaction effect: n.a.; effect group: n.s.
Wu et al. (2022)	*N* (all included) = 32 (31 active, 31 sham) *N* (completers) = 30 (15 (13f 2m) active, 15 (11f 4m) sham) Age (active, sham; mean ± SD, permitted range): 45.9 ± 15.4 years, 47.1 ± 13.2 years, 18–70 years Insomnia criteria: ICSD‐3. Additional sleep criteria: PSQI > 7	Double‐blind, randomized–controlled trial. Sham condition: transcutaneous stimulation of the periauricular area in the scapha area (sham)	taVNS	1 mA adjusted to the maximum intensity that patients could tolerate, 20 Hz, 20 min per session, 2 sessions per day, for 1 month.	Auricular concha innervated by afferent auricular branch of the vagus nerve (ABVN)	• PSQI: interaction effect: n.a.; effect group (all time points): n.s. Values (baseline, week 1, week 2, week 3, week 4; median, IQR): active: 14 (9–18), 11 (7–13), 8 (5–11), 8 (4–10.5), 6 (3–9.5) sham: 12 (8–16), 11 (6–13), 9 (5–12), 9 (5–12.5), 7 (4–11) • PSG: n.a. • Further outcome measures: HAMD: interaction effect: n.a.; effect group: n.s. HAMA: interaction effect: n.a.; effect group: n.s. “Effective rate” (number of patients with PSQI reduction by ≥ 50% from baseline): interaction effect: n.a.; effect group, week 2 n.s., week 3 n.s., week 4 ↓

Abbreviation: A, anode; BAI, Beck Anxiety Inventory; BDI, Beck's Depression Inventory; C, cathode; CBT‐I, cognitive behavioural therapy for insomnia; CI, confidence interval; DLPFC, dorsolateral prefrontal cortex; DSM, Diagnostic and Statistical Manual of mental disorders; EEG, electroencephalography; ESS, Epworth Sleepiness Scale; GAD, general anxiety disorder; HAMD, Hamilton Depression Rating Scale; HRSA, Hamilton Rating Scale for Anxiety; HRSD‐24, Hamilton Rating Scale for Depression; ICD, International Statistical Classification of Diseases and Related Health Problems; IQR, interquartile range; ISI, Insomnia Severity Index; l, left; n.a., not applicable or not reported; n.s., non‐significant; PFC, prefrontal cortex; PSG, polysomnography; PSQI, Pittsburgh Sleep Quality Index; r, right; REM, rapid eye movement; RMT, resting motor threshold; rTMS, repetitive transcranial magnetic stimulation; SAS, Self‐rating Anxiety Scale; SD, standard deviation; SDS, Self‐rating Depression Scale; SE, sleep efficiency (%); SEM, standard error of the mean; SFA, Schlaffragebogen A; SOL, sleep‐onset latency (min); tACS, transcranial alternating current stimulation; taVNS, transcutaneous auricular vagus nerve stimulation; tDCS, transcranial direct current stimulation; tES, transcranial electric stimulation; TMS, transcranial magnetic stimulation; toDCS, transcranial oscillatory direct current stimulation; TST, total sleep time (min); X → Y, vigilance state transitions from state X to state Y; WASO, wake after sleep onset (min).

^a^ Initial intention to treat sample as well as completers are reported when available. Mean, SD and range of age are reported as available. Insomnia criteria: Diagnostic manual PLUS additional sleep criteria (e.g. PSQI or ISI cut‐offs).

^b^ Conditions specified when not sham versus active. Sham condition specified.

^c^ Targeted brain region or EEG positions.

^d^ Exact values for PSQI and ISI scores are reported when provided. Interaction effect refers to [time] × [condition or group]. Pairwise comparisons refer to active versus sham post‐treatment, if not otherwise specified.

* Significant interaction or main effect (*p* < 0.05). ↑ = significant increase by pairwise comparison; ↓ = significant decrease by pairwise comparison.

Physiologically, it remains enigmatic how TMS applied to very different cortical regions and using stimulation protocols with contrary effects on cortical excitability, such as high‐ and low‐frequency rTMS of the left DLPFC, could all elicit strong sleep benefits. Several rTMS studies report strong and significant improvements not only in subjective but also in objective sleep measures, which is furthermore surprising given that the clinical presentation of insomnia is highly variable (Perlis et al., 2022) and that objective sleep disturbances in patients with insomnia are typically only moderate (Baglioni et al., 2014). This could be explained by what has been identified as substantial placebo effects for drugs and devices used for insomnia treatment (Roth et al., 2018; Winkler & Rief, 2015), which makes efficient blinding and control condition inclusion indispensable. Comparing active TMS of candidate brain regions with active TMS of cranial structures that are not part of the sleep‐regulatory system could minimize the placebo effect, clarify potential benefits of TMS for patients with insomnia, and lend to optimizing stimulation targets and protocols.

### 3.3 tES methods (tDCS and tACS)

Transcranial electrical stimulation (Figure 2b) non‐invasively modulates cortical activity by applying weak electrical currents through electrodes placed on the scalp (Stagg & Nitsche, 2011). It does not induce neuronal action potentials, but rather modifies the spontaneous activity of cortical neurons through an effective change in excitability (Ardolino et al., 2005; Zaghi et al., 2010). The most common tES techniques are tACS (alternating polarity stimulation) and tDCS (fixed polarity stimulation; Reed & Cohen Kadosh, 2018). Rhythmic stimulation using tACS or oscillatory tDCS (toDCS) enables brain activity to be modulated in a frequency‐specific manner (Fehér et al., 2017; Fröhlich & McCormick, 2010). In contrast, tDCS modulates cortical excitability in a polarity‐dependent fashion, with anodal stimulation typically increasing and cathodal stimulation decreasing cortical excitability (Nitsche & Paulus, 2000). The effects of tES are complicated; they depend heavily on the cortical target region and stimulation parameters, including stimulation current density, duration and repetition (Nitsche & Paulus, 2011). Long‐lasting neuroplastic after‐effects have been observed with prolonged stimulation (Brunoni et al., 2012; Fritsch et al., 2010), which presents the notion of developing and applying techniques to treat neurological and psychiatric conditions.

Transcranial electric stimulation has been recently explored as a tool to modulate sleep in patients with insomnia. A pilot study compared bilateral frontal cortex toDCS (0.75 Hz; 260 μA, ~25‐min stimulation duration) during the early night sleep with sham stimulation using a within‐subject design in seven patients with chronic insomnia (Saebipour et al., 2015). The authors report results from six patients who completed the study indicating that toDCS increased sleep efficiency, and suggest a sleep‐stabilizing effect of the intervention based on changes in sleep architecture. However, subjective sleep ratings did not improve significantly. Another pilot study applied tACS (0.75 mA, 0.75 Hz) over the frontal cortex in 12 patients with insomnia and reports the results of nine patients who completed the study (Motamedi et al., 2022). Stimulation began after lights were turned off as a 5‐min session while vigilance states were monitored with PSG. Another 5 min of stimulation was applied after a 1‐min break if the patient had not entered sleep. This cycle (5‐min stimulation, 1‐min break) was repeated until a sleep stage was observed via electroencephalography (EEG). The same paradigm was applied in the case of arousal lasting longer than 1 min during the night. The authors found no significant improvements in objective sleep parameters, except for a reduced arousal index following active stimulation. The authors furthermore report a lower score in the Epworth Sleepiness Scale (ESS) after active stimulation compared with sham stimulation, but only provide pre–post analysis of other sleep questionnaires without comparison to the sham condition. In both pilot studies, however, important methodological details regarding participant blinding and the design of the sham condition are missing.

A larger study in 62 patients with chronic insomnia assessed if repeated frontal cortex tACS could improve subjective sleep parameters. In contrast to the two aforementioned pilot studies, high‐frequency tACS with very strong currents was applied over 20 daily sessions (77.5 Hz, 15 mA, 40 min per session) during the day for four consecutive weeks (Wang et al., 2020). The authors report that, when compared with the sham group, the active group demonstrated significant improvements of insomnia symptoms immediately at the end of the 4‐week intervention as well as at the follow‐up appointment 4 weeks post‐intervention. In particular, the study reports lower PSQI scores as well as subjectively shorter SOL, increased TST, greater sleep efficiency (SE) and better sleep quality in the active stimulation group compared with that of the control. However, it must be highlighted that the applied current density was several times higher than that which is used in conventional tES studies and falls above the safety limits of 2–4 mA stated in international safety and ethics guidelines (Antal et al., 2017). The study reports epileptiform discharges in several patients over the 8‐week observation period, which should raise health concerns regarding the stimulation parameters.

Instead of superimposing brain oscillations, another line of tES research entails counteracting cortical hyperarousal by modulating cortical excitability. To this aim, Frase and colleagues applied bifrontal anodal, cathodal and sham tDCS in a within‐subject design involving 19 patients with clinically‐diagnosed insomnia. tDCS was applied immediately prior to nighttime sleep with the hypothesis that anodal stimulation would shorten TST and cathodal stimulation prolong TST (Frase et al., 2019). However, the authors found no difference in TST between stimulation conditions. They report no differences in PSG sleep continuity, measures of sleep architecture or subjective sleep parameters between control and sham groups.

Shifting focus towards patients with comorbid insomnia and major depressive disorder (MDD), a double‐blinded study randomized 90 patients to an active or sham tDCS group (Zhou et al., 2020). The tDCS procedure included 20 sessions (2 mA; 30 min each) of left anodal and right cathodal DLPFC stimulation over 4 weeks. The authors report a greater improvement in depression and anxiety scores, a greater increase of PSG‐recorded TST, and a stronger improvement of objective and subjective sleep efficiency in the active tDCS compared with that of the sham condition. Importantly, no other subjective or objective sleep parameters – including the PSQI – showed a significant interaction effect between time and active/sham condition. While main and exploratory analyses provide some indication that tDCS protocols might improve sleep in patients with insomnia with depression, the presentation of the results is inconsistent. Some reported interaction effects might be furthermore confounded by numerically higher baseline values in the active tDCS group, by changes in depression symptomatology or by medication intake.

In summary, five studies have used tES to modulate and alter brain activity in patients with insomnia (Table 1). One study applied stimulation during sleep (Saebipour et al., 2015), while the others applied stimulation during the process of falling asleep (Motamedi et al., 2022), immediately prior to sleep (Frase et al., 2019) or during wakefulness with repeated stimulation sessions (Wang et al., 2020; Zhou et al., 2020). tES targeted the left DLPFC (Zhou et al., 2020) and the PFC, with different electrode arrangements including bilateral Fp1/Fp2 (Frase et al., 2019), bilateral F3/F4 (Motamedi et al., 2022), bilateral F3/F4 (Saebipour et al., 2015), and single electrode configurations over Fpz, Fp1 and Fp2 (Wang et al., 2020). Most studies (four out of five) reported favourable results that indicate superiority of active tES stimulation compared with the sham condition. However, there was a notable risk of bias in all studies resulting from insufficient or unclear blinding, non‐transparent sham conditions and inconsistent statistical reporting (Figure S1). Furthermore, two pilot studies presented sample sizes of less than 10 participants, and one study exceeded the internationally recognized safety limits. This highlights the need for future well‐controlled trials using established stimulation protocols.

### 3.4 Vagus nerve stimulation

The vagus nerve is the 10th cranial nerve, and consists of afferent fibres carrying somatic and visceral information to the brain, and efferent fibres that mostly modulate the activity of visceral organs as part of the parasympathetic nervous system. Invasive stimulation has been long established as a treatment for drug‐resistant epilepsy and depression, and can be achieved by implanting and typically wrapping a fine wire electrode around the left cervical vagus nerve (Johnson & Wilson, 2018). However, non‐invasive transcutaneous electrical stimulation of the cervical or auricular branch can elicit manifold central and peripheral effects (Badran et al., 2018; Butt et al., 2020). taVNS (Figure 2c) targets the only peripheral branch of the vagus nerve by applying electrical stimulation to the tragus or concha of the ear (Kaniusas et al., 2019). Therefore, taVNS is currently being tested as an attractive non‐invasive treatment option for epilepsy, depression and an increasing range of psychiatric conditions.

Two trials from the same institution have systematically studied the effects of taVNS in patients with insomnia (Jiao et al., 2020; Wu et al., 2022). Both compared stimulation of the auricular concha against stimulation of the auricular scapha, which is not innervated by the auricular branch of the vagus nerve and therefore serves as an effective control (Table 1). In the first study, 72 patients received two sessions of 20‐Hz dilatational wave stimulation twice a day, for 5 days a week over 2 weeks (Jiao et al., 2020). While PSQI scores and questionnaire evaluation of sleepiness, fatigue, depression and anxiety were shown to significantly improve in both groups, no differences were reported between active and control conditions. The second study used a similar stimulation protocol with 20‐Hz stimulation twice a day over 1 month (Wu et al., 2022). Though discontinued, an interim analysis of the primary outcome parameter “effective rate” – defined as ≥ 50% reduction of PSQI scores from baseline – presents significantly greater outcomes in the treatment than in the control group when considering data from the first 32 patients. However, no significant group difference was observed in PSQI scores or any secondary outcome parameter.

In summary, although various studies using a pre–post design without a control condition report strong effects of taVNS on insomnia symptoms (He et al., 2022; Wu et al., 2021; Zhang et al., 2021), current evidence from sham‐controlled trials does not indicate substantial effects of taVNS on improving insomnia symptoms.

### 3.5 Forehead cooling

Forehead cooling techniques are based on the notion that lowering cranial temperature decreases brain metabolism (Erecinska et al., 2003). Elevated metabolic activity in the frontal cortex during sleep is thought to contribute to sleep fragmentation and enhanced wake perception of patients with insomnia (Figure 2d; Nofzinger et al., 2004, 2006); therefore, forehead cooling aims to counteract this effect.

One randomized–controlled trial compared a forehead cooling device against a sham condition in 106 patients with insomnia (Roth et al., 2018). The sham condition consisted of an inactive vestibular stimulation apparatus that served as a credible device control. It was presented to both the patients and technical staff conducting the study as an active treatment condition and therefore appropriately established a double‐blinded study design. Forehead cooling was performed with a urethane forehead bladder at an adjustable temperature between 14 and 16°C. No significant differences were observed for latency to persistent sleep and absolute sleep efficiency in active cooling versus sham conditions. Similarly, no significant effects were reported for any subjective sleep parameters. Nevertheless, upon more detailed investigation, the authors reported a significantly greater reduction in sleep‐state‐specific onset latency and a greater amount of sleep during the first hour of the night in the treatment condition compared with the control. The authors concluded that frontal cerebral thermal therapy facilitates sleep induction in patients with insomnia. However, they also very importantly highlight that, in this large and well‐controlled trial, the credible sham device condition elicited strong pre–post effects with significant improvements in subjective and objective sleep parameters (Roth et al., 2018). The authors discuss that the effect of this sham device was larger than the known placebo effects of control conditions in pharmacotherapy and CBT‐I trials. This finding illustrates that a reliable sham condition is always required. Consequently, caution is warranted when interpreting pre–post improvements across subjective and objective sleep parameters as evidence for the efficacy of any brain stimulation device.

## 4 DISCUSSION

The exploration of brain stimulation approaches as potential treatment for insomnia is currently seeing a renaissance following historical attempts to electrically improve sleep of patients with insomnia in the 19th century (for a review, see Wagner & Steinberg, 2021) and trials on “electrosleep” therapy for insomnia occurring in the mid‐20th century (Frankel et al., 1973; Weiss, 1973). Modern NIBS techniques have well‐established effects on neurophysiology, fuelling new hopes that brain stimulation could finally prove beneficial in treating insomnia. Most of the trials included in this review were published in the past 3 years, and used TMS or tES. These techniques modulate cortical activity, and are already used as treatment options for specific neurological and psychiatric conditions. While most of the TMS and tES trials included in this review report a striking improvement in subjective and objective sleep parameters, all have considerable methodological limitations. It remains a mystery as to how stimulating diverse cortical targets – at variable times of the day/night and with considerably different stimulation techniques and parameters – could all induce similarly strong treatment effects. Some enthusiastic reports are reminiscent of the initial excitement surrounding “electrostatic showers”, “faradisation”, “teslaisation” and the initial praise of electrosleep treatment for insomnia. Each later failed to prove effective in systematic trials (Templer, 1975; Wagner & Steinberg, 2021). Importantly, the only two studies we presently reviewed that demonstrate a low risk of bias and credible device controls reported strong pre–post effects of NIBS on subjective and even objective PSG‐measured sleep parameters in active and control conditions, but no differences in the primary outcomes between groups (Jiao et al., 2020; Roth et al., 2018). This indicates that brain stimulation devices elicit strong placebo effects and that, therefore, flawless sham conditions and blinded study designs are critical when testing and evaluating potential therapeutic benefits.

### 4.1 Mechanistic considerations

The hyperarousal model of insomnia postulates that cortical hyperactivity during sleep contributes to the sleep disturbances (Riemann et al., 2010). This might be reflected both in increased brain metabolism and in increased high‐frequency oscillatory activity during sleep. Therefore, a reduction of cortical excitability or superimposing slow rhythms presents as a logical treatment approach. However, attempts to characterize consistent patterns of functional disturbances in patients with insomnia using neuroimaging have overall been inconclusive, likely due to heterogeneity of patient populations (Tahmasian et al., 2018). Heterogeneity in cortical metabolic signatures must also be considered as, for example, an influential study that reported generally increased brain metabolism in patients with insomnia also finds hypometabolism in individual brain regions including the PFC specifically during wakefulness (Nofzinger et al., 2004). Therefore, stimulation effects on sleep likely depend on the time of day and ongoing brain activity.

### 4.2 Insights from basic research

Constantin von Economo, who provided a first localization of sleep–wake areas in the human brain, expressed the hope that *“the exact knowledge of the localization of the center for sleep regulation […], would make it possible to treat insomnia and other sleep disturbances in a better and more active way […]”* (Economo, 1930). Thanks to recent technological advances, basic research is now rapidly clarifying the contribution of several brain areas to sleep–wake regulation (Saper & Fuller, 2017). While the cerebral cortex is traditionally not considered relevant for vigilance state control, an important role for the cerebral cortex in sleep–wake regulation has recently been shown in mice (Krone et al., 2021) with the PFC specifically contributing to sleep‐preparatory behaviour and initiation (Tossell et al., 2020). In addition, the predominance of frontal cortical area activity during the build‐up of sleep debt (Cajochen et al., 1999; Werth et al., 1996), alongside the initiation and synchronization of slow oscillations and sleep spindles (Marzano et al., 2013; Massimini et al., 2004), renders this region a promising target for sleep modulation approaches. Research in healthy volunteers suggests that sleep modulation via tDCS of frontal cortical regions is possible (Frase et al., 2016), which supports a proposal of a top‐down control mechanism governing arousal and sleep (Krone et al., 2017). Growing insights into sleep physiology reinforce the hope that exact knowledge surrounding brain structures and activity patterns that govern vigilance state control will facilitate the development of effective sleep modulation therapies.

### 4.3 Alternative stimulation approaches

In addition to the aforementioned brain stimulation approaches, for which clinical trials on patients with insomnia have already been published, several other approaches are being investigated for their potentially beneficial sleep‐modulatory properties.

**FIGURE 3:**
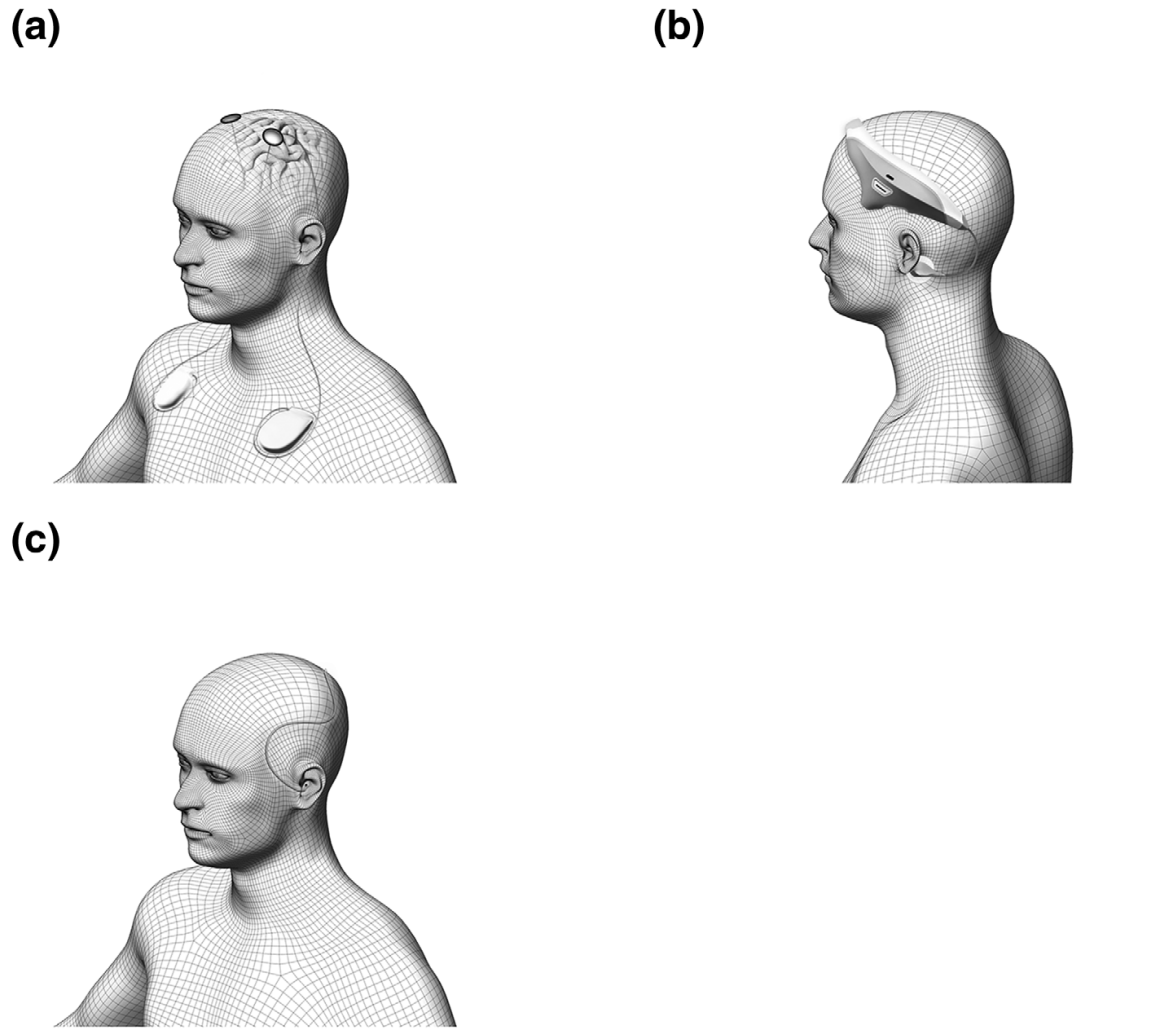
Brain stimulation techniques with potential applications in insomnia treatment: (a) deep brain stimulation (DBS); (b) vestibular nerve stimulation (VeNS); (c) auditory stimulation.

*Deep brain stimulation* involves electrically stimulating circumscribed brain regions after the neurosurgical implantation of a fine electrode (Figure 3a). This stimulation technique has seen great success in treating movement disorders – particularly in patients with Parkinson's disease. However, its use in treating psychiatric conditions remains limited due to great heterogeneity in clinical presentation, a lack of clear pathophysiological models and subsequent difficulty in selecting efficient stimulation targets (Lozano et al., 2019). Several DBS studies have monitored sleep as a secondary outcome parameter, yet have yielded highly variable results (Baumann‐Vogel et al., 2017; Cavalloni et al., 2021). To our knowledge, no study has yet investigated the potential of DBS as a treatment for primary insomnia. However, the lack of an unambiguous candidate region as well as the ethical constraints related to the small but serious risk of surgical complications make it unlikely that DBS will soon be used in the treatment of insomnia.

*Transcutaneous vestibular nerve stimulation* (*VeNS*) and bed rocking are two methods to stimulate the vestibular apparatus, which is the motion‐sensing structure located in the inner ear (Figure 3b). Some evidence suggests that stimulating the vestibular system using rocking movements can affect sleep architecture, brain oscillations and sleep‐related memory consolidation both in animals and in healthy human participants (Bayer et al., 2011; Kompotis et al., 2019). However, conflicting results have been reported regarding the effectiveness of this technique in modulating sleep (Omlin et al., 2018; van Sluijs et al., 2020). A pilot study using VeNS prior to nighttime sleep in patients with insomnia reported improvements of subjective sleep parameters, yet without comparison to a control group or condition (Goothy & McKeown, 2021).

*Auditory stimulation* (Figure 3c) has been used since the early days of sleep research to probe arousal thresholds (Blake & Gerard, 1937; Neckelmann & Ursin, 1993) or to disrupt slow‐wave sleep (Dijk et al., 1987). However, a decade ago, it was found that applying closed‐loop auditory stimuli during sleep can enhance slow oscillations, sleep spindles and sleep‐dependent memory consolidation (Ngo et al., 2013). Since then, physiological effects of various auditory stimulation paradigms on oscillatory brain activity and subsequent molecular, network and behavioural effects have been demonstrated (Fattinger et al., 2017; Lustenberger et al., 2018). While most research to date has been dedicated to developing the technology to elicit robust stimulation effects on sleep oscillations and sleep‐related functions, modulated sleep architecture has also been recently demonstrated (Fehér et al., 2023). Several trials are now underway that aim to explore the potential of auditory stimulation paradigms in improving insomnia symptoms.

### 4.4 Improving daytime function

The diagnosis of insomnia requires at least one daytime symptom such as fatigue, impaired concentration or daytime sleepiness, but is independent of objectifiable sleep disruption (Riemann et al., 2017). Surprisingly, many brain stimulation trials have focused on improving objective sleep parameters, which is a difficult undertaking given the heterogeneity of PSG findings in patients with insomnia (Baglioni et al., 2014). TMS and tDCS daytime protocols have demonstrated small but robust effects on working memory and attention across brain disorders (Begemann et al., 2020). tDCS has additionally been shown to mitigate the daytime effects of sleep deprivation in healthy volunteers (Alfonsi et al., 2023). In addition, cognitive workload and daytime activity likely increase sleep pressure (Goel et al., 2014), suggesting that improved daytime function might indirectly benefit sleep. We therefore propose that improving daytime function through brain stimulation techniques should be tested as an alternative therapeutic strategy.

## 5 CONCLUSION

Researchers and clinicians now possess the ability to modulate brain activity with increasing efficacy using modern brain stimulation techniques. However, despite many reports that brain stimulation improves subjective and objective sleep parameters, well‐controlled studies indicate that sham stimulation devices have similarly strong and robust effects. To date, no brain‐stimulation protocol exists that could claim relevant therapeutic benefits for insomnia. While animal and human research clearly indicates that sleep modulation through brain stimulation is possible, clinical translation is hampered by gaps in our current understanding of sleep physiology and insomnia pathophysiology. As most methods modulate cortical activity, understanding how the cortex contributes to sleep regulation and which patterns of cortical activity are altered in patients with insomnia will be essential when optimizing stimulation targets and protocols. Auditory stimulation provides a novel approach to sleep modulation, and first results from clinical trials in patients with insomnia are soon to be expected. Improving daytime function might be an alternative or additional strategy in applying brain‐stimulation tools to insomnia treatment. Informed by the studies presented in this systematic review, we provide action points to support the reliable and effective design of future clinical trials investigating the use of brain‐stimulation techniques in the treatment of insomnia.

Action points

- Methodological improvements:
  - Implement double‐blinding and test for successful blinding;
  - Standardize sham protocols that are indistinguishable from active stimulation;
  - Indicate the precise time of day as to when stimulation is performed (e.g. morning, during the day, prior to sleep or during sleep).
- Stimulation based on mechanistic insights:
  - Select stimulation targets that are part of known sleep‐regulating circuitry (informed by brain imaging, animal research or successful sleep manipulation in healthy subjects);
  - Consider the time of day and duration of stimulation effects (e.g. excitation in the morning to improve daytime symptoms, reduction of arousal in the evening, and oscillatory stimulation during sleep to mimic typical sleep oscillations).
- Patient selection based on study aim:
  - Chose patient populations according to stimulation methods and outcome measures (e.g. assess objective sleep measures in patients with insomnia with objective sleep disruption, assess effects of stimulation to improve alertness in patients with disrupted daytime function, and assess methods for sleep initiation in patients with difficulties falling asleep);
  - Consider that the effects on sleep in patients with comorbidities might be mediated by changes in the comorbid condition;
  - Avoid psychopharmacology due to confounding effects on sleep and stimulation effectiveness.
- Optimize selection and reporting of outcome parameters:
  - Use standardized sleep questionnaires (e.g. PSQI or ISI) when assessing subjective sleep quality;
  - Report standard variables, if PSG is performed, to avoid selectively reporting positive results;
  - Discuss interaction effects of “time” and “group”, and avoid overinterpreting pre–post effects that might be due to strong placebo effects of stimulation devices.

## AUTHOR CONTRIBUTIONS

**Lukas B. Krone:** Conceptualization; funding acquisition; writing – original draft; methodology; validation; writing – review and editing; project administration; supervision; data curation; investigation; formal analysis; resources. **Kristoffer D. Fehér:** Conceptualization; investigation; writing – original draft; methodology; validation; visualization; writing – review and editing; formal analysis; data curation. **Tania Rivero:** Methodology; validation; software; formal analysis; data curation; writing – review and editing; writing – original draft; resources. **Ximena Omlin:** Conceptualization; investigation; writing – original draft; methodology; validation; writing – review and editing; formal analysis; data curation; supervision.

## CONFLICT OF INTEREST

All authors declare no conflict of interest.

## Supporting information


Supplementary Figure S1.

Supplementary File S1.

## Data Availability

The complete list of search results is reproducible using the search strategy listed in Supplementary File 1. The list is also available upon request.
