# Supplementary material for: Brain stimulation techniques as novel treatment options for insomnia: A systematic review
Source: J Sleep Res. 2023 May 18;32(6):e13927. doi: 10.1111/jsr.13927 (PMC10909439; doi:10.1111/jsr.13927)
Supplement: Supplementary file 1 — Supplementary Figure S1. [file JSR-32-e13927-s002.pdf]

Risk of Bias Assessment

Study	Intervention	D1	D2	D3	D4	D5	Overall
Jiang et al. 2013	rTMS	+	!	+	+	-	-
Huang et al. 2018	rTMS	+	+	+	!	+	!
Zhang et al. 2018	rTMS	+	!	+	-	!	-
Zhang et al. 2022	rTMS	+	!	+	+	-	-
Li et al. 2022	rTMS	+	!	+	!	-	-
Lu et al. 2022	rTMS	+	-	+	!	-	-
Guo et al. 2023	rTMS	+	!	+	!	-	-
Zhang et al. 2023	rTMS	+	!	+	!	-	-
Pu et al. 2023	rTMS	+	-	+	!	+	-
Saebipour et al. 2015	toDCS	!	!	+	-	-	-
Frase et al. 2019	tDCS	+	!	+	+	+	!
Zhou et al. 2020	tDCS	+	+	+	+	!	!
Wang et al. 2020	tACS	+	+	+	+	!	!
Motamedi et al. 2022	tACS	+	!	!	-	-	-
Roth et al. 2018	Forehead cooling	+	+	+	+	+	+
Jiao et al. 2020	taVNS	+	+	+	+	+	+
Wu et al. 2022	taVNS	+	+	+	+	!	!

+ Low risk

! Some concerns

- High risk

D1 Randomisation process

D2 Deviations from the intended interventions

D3 Missing outcome data

D4 Measurement of the outcome

D5 Selection of the reported result

rTMS: Repetitive transcranial magnetic stimulation

tDCS: Transcranial direct current stimulation

toDCS: Transcranial oscillatory direct current stimulation

tACS: Transcranial alternating current stimulation

taVNS: Transcutaneous auricular vagus nerve stimulation
