# Supplementary material for: Brain stimulation techniques as novel treatment options for insomnia: A systematic review
Source: J Sleep Res. 2023 May 18;32(6):e13927. doi: 10.1111/jsr.13927 (PMC10909439; doi:10.1111/jsr.13927)
Supplement: Supplementary file 2 — Supplementary File S1. [file JSR-32-e13927-s001.docx]

**MEDLINE (R) ALL (Ovid),** Coverage 1946-Present

1 Electric Stimulation/ or deep brain stimulation/ or transcranial direct current stimulation/ or Transcutaneous Electric Nerve Stimulation/ or vagus nerve stimulation/ or Transcranial Magnetic Stimulation/ or Acoustic Stimulation/

2 (((deep brain* or DBS or brain or vagal nerve* or vagus nerve* or transcranial* or transcutaneous* or vestibular* or anodal* or cathodal* or direct current* or alternating current* acoustic* or auditory* or ultrasound* or binaural* or thermal or temperature) adj3 stimulation*) or ((brain or forehead or head or cerebral) adj2 (cooling or temperature or thermal)) or white noise or brown noise or pink noise or green noise or broadband noise or broadband sound or noise masking or sound masking or auditory masking or masking noise or masking sound or TMS or tdcs or tdcss or rTMS or tES or tACS).ti,ab.

3 1 or 2

4 "Sleep Initiation and Maintenance Disorders"/ or Sleep Wake Disorders/

5 insomni*.ti,ab.

6 4 or 5

7 3 and 6

8 (exp animals/ or animal experimentation/ or models, animal/ or exp plants/ or exp fungi/) not humans/

9 (mouse or mice or rat or rats or animal or animals or cell?).ti.

10 or/8-9

11 7 not 10

**Embase (Ovid),** Coverage 1947-Present

1 vestibular stimulation/ or electrostimulation/ or brain depth stimulation/ or transcranial electrical stimulation/ or transcranial direct current stimulation/ or transcranial alternating current stimulation/ or transcutaneous electrical nerve stimulation/ or vagus nerve stimulation/ or transcranial magnetic stimulation/ or transcranial random noise stimulation/ or auditory stimulation/ or auditory masking/ or white noise/ or brain temperature/

2 (((deep brain* or DBS or brain or vagal nerve* or vagus nerve* or transcranial* or transcutaneous* or vestibular* or anodal* or cathodal* or direct current* or alternating current* acoustic* or auditory* or ultrasound* or binaural* or thermal or temperature) adj3 stimulation*) or ((brain or forehead or head or cerebral) adj2 (cooling or temperature or thermal)) or white noise or brown noise or pink noise or green noise or broadband noise or broadband sound or noise masking or sound masking or auditory masking or masking noise or masking sound or TMS or tdcs or tdcss or rTMS or tES or tACS).ti,ab.

3 1 or 2

4 insomnia/ or primary insomnia/

5 insomni*.ti,ab.

6 4 or 5

7 3 and 6

8 (exp animal/ or exp invertebrate/ or nonhuman/ or animal experiment/ or animal tissue/ or animal model/ or exp plant/ or exp fungus/) not (exp human/ or human tissue/)

9 (mouse or mice or rat or rats or animal or animals or cell?).ti.

10 or/8-9

11 7 not 10

**APA PsycINFO (Ovid),** Coverage, 1806-Present

1 electrical stimulation/ or brain stimulation/ or electrical brain stimulation/ or deep brain stimulation/ or transcranial direct current stimulation/ or vagus nerve/ or transcranial magnetic stimulation/ or auditory stimulation/ or auditory masking/ or white noise/

2 (((deep brain* or DBS or brain or vagal nerve* or vagus nerve* or transcranial* or transcutaneous* or vestibular* or anodal* or cathodal* or direct current* or alternating current* acoustic* or auditory* or ultrasound* or binaural* or thermal or temperature) adj3 stimulation*) or ((brain or forehead or head or cerebral) adj2 (cooling or temperature or thermal)) or white noise or brown noise or pink noise or green noise or broadband noise or broadband sound or noise masking or sound masking or auditory masking or masking noise or masking sound or TMS or tdcs or tdcss or rTMS or tES or tACS).ti,ab.

3 1 or 2

4 insomnia/

5 insomni*.ti,ab.

6 4 or 5

7 3 and 6

8 (mouse or mice or rat or rats or animal or animals or cell?).ti.

9 7 not 8
